# Facilitating harmonized data quality assessments. A data quality framework for observational health research data collections with software implementations in R

**DOI:** 10.1186/s12874-021-01252-7

**Published:** 2021-04-02

**Authors:** Carsten Oliver Schmidt, Stephan Struckmann, Cornelia Enzenbach, Achim Reineke, Jürgen Stausberg, Stefan Damerow, Marianne Huebner, Börge Schmidt, Willi Sauerbrei, Adrian Richter

**Affiliations:** 1 Institute for Community Medicine, Department SHIP-KEF, University Medicine Greifswald, Greifswald, Germany; 2 Institute for Medical Informatics, Statistics, and Epidemiology, University of Leipzig, Leipzig, Germany; 3 Leibniz Institute for Prevention Research and Epidemiology – BIPS, Bremen, Germany; 4 Institute for Medical Informatics, Biometry and Epidemiology (IMIBE), Faculty of Medicine, University of Duisburg-Essen, Duisburg, Germany; 5 Robert Koch Institute, Department of Epidemiology and Health Monitoring, Berlin, Germany; 6 Department of Statistics and Probability, Michigan State University, East Lansing, MI USA; 7 Institute of Medical Biometry and Statistics, Faculty of Medicine and Medical Center, University of Freiburg, Freiburg, Germany

**Keywords:** Data quality, Observational health studies, Data quality indicators, Data quality monitoring, Initial data analysis, R

## Abstract

**Background:**

No standards exist for the handling and reporting of data quality in health research. This work introduces a data quality framework for observational health research data collections with supporting software implementations to facilitate harmonized data quality assessments.

**Methods:**

Developments were guided by the evaluation of an existing data quality framework and literature reviews. Functions for the computation of data quality indicators were written in R. The concept and implementations are illustrated based on data from the population-based Study of Health in Pomerania (SHIP).

**Results:**

The data quality framework comprises 34 data quality indicators. These target four aspects of data quality: compliance with pre-specified structural and technical requirements (*integrity*); presence of data values (*completeness*); inadmissible or uncertain data values and contradictions (*consistency*); unexpected distributions and associations (*accuracy*). R functions calculate data quality metrics based on the provided study data and metadata and R Markdown reports are generated. Guidance on the concept and tools is available through a dedicated website.

**Conclusions:**

The presented data quality framework is the first of its kind for observational health research data collections that links a formal concept to implementations in R. The framework and tools facilitate harmonized data quality assessments in pursue of transparent and reproducible research. Application scenarios comprise data quality monitoring while a study is carried out as well as performing an initial data analysis before starting substantive scientific analyses but the developments are also of relevance beyond research.

## Background

Achieving a high data quality is a precondition for valid research results in all empirical sciences. Informative data quality indicators should inform data analysts about the “degree to which a set of inherent characteristics of data fulfils requirements” (ISO 8000). Data quality indicators thus describe actual and potential deviations from defined requirements such as formal compliance with pre-specified data structures, completeness, and the correctness of data values. Appropriately designing, assessing and quantifying data quality is of relevance during the entire research data life cycle. Already before the start of a data collection, having a clear understanding of data quality and its assessment should influence study design and data management. During study conduct, results of data quality assessments inform about the successful implementation of examinations, thereby triggering quality control and quality assurance activities such as data cleaning or training measures [1]. Data quality assessments after the end of a data collection influence decisions about data pooling and data harmonization [2], they can be used to benchmark studies and are necessary to safeguard responsible statistical analysis [3, 4].

While many data quality frameworks exist in the medical sciences [5–16], most of them target registries and electronic health records (EHR). These use data that have been generated outside of a research context, e.g. from administrative data. Yet, there is insufficient guidance on conducting data quality assessments for data that have specifically been generated for observational health research.

This lack of guidance is problematic as data quality frameworks for EHR data and registries are not directly applicable to designed research data collections [17]. For example, *accessibility* and *interpretability* have been defined as major quality criteria for EHR data [16]. Both are less relevant in research data collections where related issues are commonly solved by an appropriate study design, the standardisation of procedures, the training of examiners, and the implementation of a supporting infrastructure. Furthermore, preconditions for the computation of indicators may differ. Calculating the exact proportion of missing data in a population-based cohort study is based on a known sampling frame with a precisely defined number of study variables for each participant. In contrast, if, for example, information on a defined cardiovascular comorbidity in a patient with diabetes is missing in an EHR data set it is commonly unclear whether this comorbidity has not been diagnosed, examined, or simply not recorded. Therefore, a data quality framework must take specifics of the targeted data body into account.

A data quality framework must also guide the use of metadata and process variables for data quality assessments. Metadata in this context refers foremost to attributes that describe variables and expected data properties such as admissible values or distributional properties. Process variables describe aspects of the data generating process such as time stamps, observers or devices. Process variables are used to detect unexpected associations with study outcomes of interest. Ideally, each data quality indicator is accompanied by a description of the metadata and process variables that are required for its computation.

While a growing number of statistical routines address data quality issues [18–21], particularly in the programming language R [22–24], these routines are mostly not founded in data quality frameworks. Exceptions for EHR data are the approaches of Kahn et al. [10] within OHDSI [25] and Kapsner et al. [26].

The objectives of this work are twofold: (1) to provide a data quality framework tailored for designed data collections in observational health research, (2) to ease the application of the framework by providing openly available software implementations. All developments were integrated in a web-page to facilitate their successful application.

## Methods

### Background

We built on an existing data quality framework, the 2nd edition of the TMF (Technology, Methods, and Infrastructure for Networked Medical Research) guideline for data quality [11, 14]. TMF is a major umbrella organization for networked medical research in Germany. The guideline was chosen because, unlike other frameworks, it includes data quality indicators, which are of specific relevance for cohort studies. Literature reviews and overviews of data quality concepts in health research [5–10, 27, 28] informed the development of our framework.

The focus of the presented framework is “intrinsic data quality” [16] which means that “data have quality in their own right”. Evaluating intrinsic data quality rests primarily on knowledge about the data generating process. This is in contrast to “contextual data quality” which means that data quality is considered within the context of a particular task, e.g. the analysis of a defined scientific research question. We currently exclude such task- and situation-specific indicators.

### Evaluation of the TMF guideline for data quality

The TMF guideline for data quality was subject to an evaluation by representatives of German general-population cohort studies to assess its suitability for this study type. Details of the evaluation process and results are available elsewhere [29]. In total, 43 out of the 51 quality indicators in the guideline have been assessed as being potentially relevant for cohort studies. In total 29 were classified as essential or important (mean evaluation score < =2; out of: 1 = essential, 2 = important, 3 = less important, and 4 = not important) and have been included in the current framework. Metrics of data quality indicators in the TMF guideline are restricted to counts and percentages, yet a broader scope of statistical metrics related to distributions, associations and measures of agreement were considered important for the quantification of aspects of data quality, as was a more specific handling of metadata compared to the TMF guideline. Therefore, novel indicators that cover aspects of descriptive statistics and initial data analysis [3] were added.

### Computing data quality with R

Functions were developed as part of this project in the *dataquieR* package, available at CRAN [30], to compute data quality indicators, using R as the programming language because of its widespread use and free access [31]. We followed the style guide first published by Hadley Wickham [32]. R scripts were tested on simulated data and on data from several cohort studies, e.g. Study of Health in Pomerania [33], LIFE-Adult-Study [34], and the IDEFICS study [35]. An R Markdown generated website provides access to the concept, dataquieR functions, sample data, metadata descriptions, references, and tutorials [36].

### Application example

The framework and implementations are illustrated using data from the Study of Health in Pomerania (SHIP), a population-based cohort study [33]. We used data from the baseline assessment of SHIP-0 from 1997 to 2001 (*N* = 4308). The data set comprises variables on: height, weight, and waist circumference from the somatometric examination, systolic and diastolic blood pressure from a blood pressure measurement, and information on smoking, marital status and intake of contraceptives from the computer assisted medical interview. An anonymized dataset was created based on a 50% random subset of the original sample (*N* = 2154). It is publicly available at [36].

R Markdown reports were rendered to HTML documents. These provide an overview of the results of the data quality assessment, including tables, and graphs. Modified study data sets are automatically generated to highlight unexpected findings at the level of individual observations with the purpose of simplifying subsequent data management steps.

## Results

### Structure of the data quality framework

In accordance with existing data quality concepts [6, 7, 9], *completeness* and *correctness* are the two core aspects of data quality (Table 1). *Completeness* is represented as a single dimension while *correctness* is subdivided into the two dimensions *consistency* and *accuracy*. The reason for this separation is introduced in the paragraph *correctness*. A precondition for successfully conducting any data quality assessment is the correct technical setup of study data and metadata. Related aspects are targeted within the *integrity* dimension.

**Table 1:** Data Quality Dimensions and Domains

Name Dimension Domain	Definition	Primary reference objects to detect data quality issues	Primary reporting metrics of indicators
**Integrity**	The degree to which the data conforms to structural and technical requirements.		
Structural data set error	The observed structure of a data set differs from the expected structure.	Data elements, data records	N
Relational data set error	The observed correspondence between different data sets differs from the expected correspondence.	Data sets	N
Value format error	The technical representation of data values within a data set does not conform to the expected representation.	Data fields	N, %
**Completeness**	The degree to which expected data values are present.		
Crude missingness	Metrics of missing data values that ignore the underlying reasons for missing data.	Data fields	N,%
Qualified missingness	Metrics of missing data values that use reasons underlying missing data.	Data fields, data elements, data record	N,%
**Consistency**	Consistency		
Range and value violations	Observed data values do not comply with admissible data values or value ranges.	Data fields	N,%
Contradictions	Observed data values appear in impossible or improbable combinations.	Data fields	N,%
**Accuracy**	The degree of agreement between observed and expected distributions and associations.		
Unexpected distributions	Observed distributional characteristics differ from expected distributional characteristics.	Data elements, data records	Diverse statistical measures^a^
Unexpected associations	Observed associations differ from expected associations.	Data elements, data records	Diverse statistical measures^a^
Disagreement of repeated measurements	Disagreement between repeated measurements of the same or similar objects under specified conditions.	Data elements, data records	Diverse statistical measures^a^

N: number of issues; %: the percentage of issues relative to the number of assessed elements in a data structure

^a^ A wide range of statistical metrics may apply such as location, scale or shape parameters, correlation coefficients, measures of agreement

**Fig. 1:**
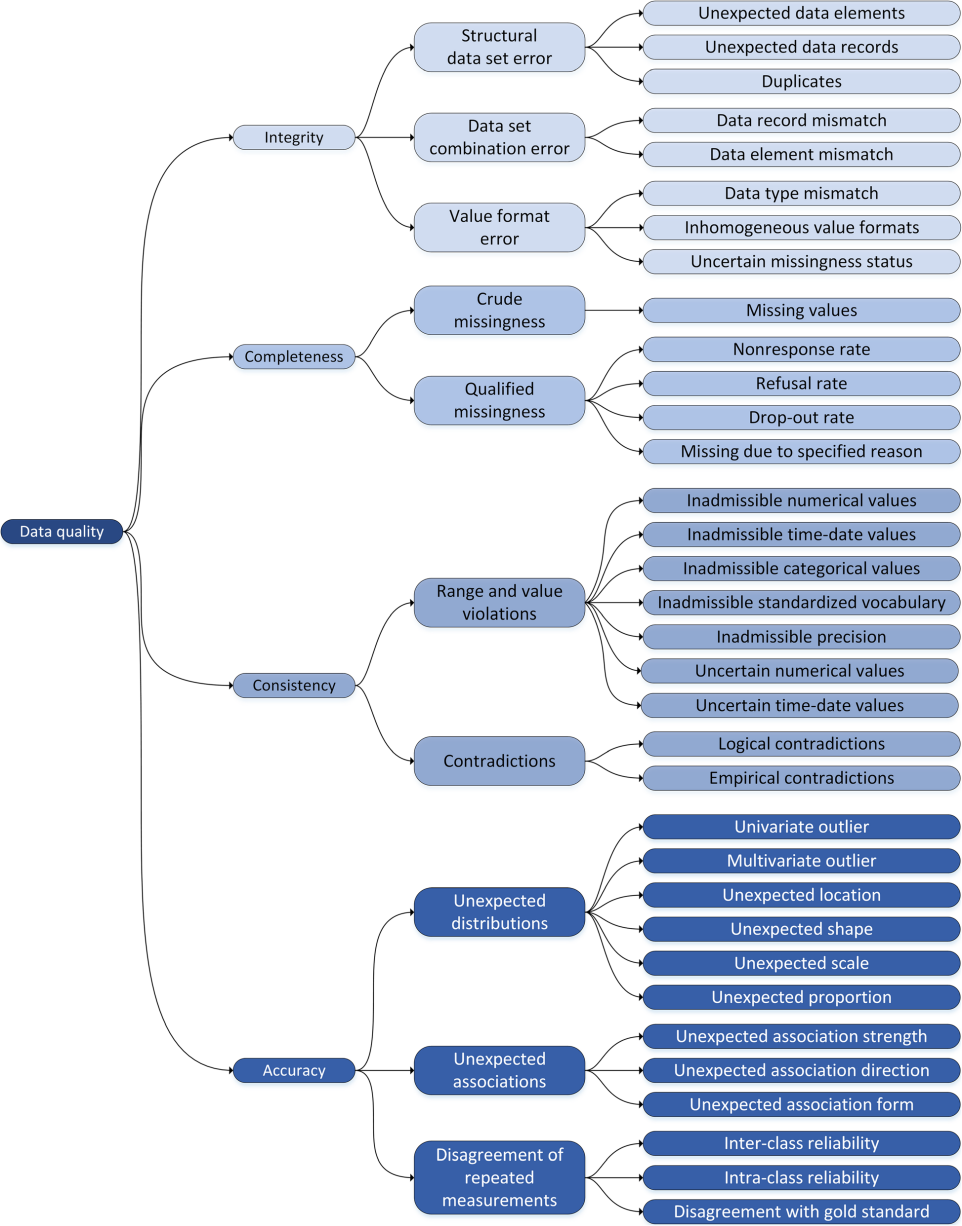
Data Quality Concept Overview

**Fig. 2:**
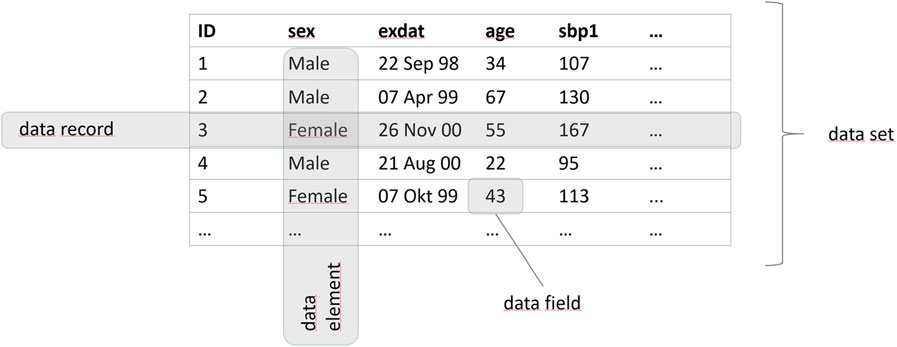
Key terms related to data structures

Each dimension is subdivided into different *data quality domains*, an overview on dimensions and domains is provided in Table 1. The domains differ mainly in terms of the methodology used to assess data quality. The next level defines *data quality indicators* (Table 2). Currently, 34 indicators are distinguished. They describe quality attributes of the data at the level of single data fields, data records, data elements, and data sets [37]. Figure 1 displays the hierarchical structure. Figure 2 illustrates the used nomenclature of terms for data structures within the framework.

**Table 2:** Overview on Data Quality Indicators with Definitions

ID	Name of indicator	Definition
Integrity		
DQI-1001	Unexpected data elements	The observed set of available data elements does not match the expected set.
DQI-1002	Unexpected data records	The observed set of available data records does not match the expected set.
DQI-1003	Duplicates	The same data elements or data records appear multiple times.
DQI-1004	Data record mismatch	Data records from different data sets do not match as expected.
DQI-1005	Data element mismatch	Data elements from different data sets do not match as expected.
DQI-1006	Data type mismatch	The observed data type does not match the expected data type.
DQI-1007	Inhomogeneous value formats	The observed data values have inhomogeneous format across different data fields.
DQI-1008	Uncertain missingness status	System indicated missing values (e.g. NA/./Null …) appear where a qualified missing code is expected.
Completeness		
DQI-2001	Missing values	Data fields without a measurement value.
DQI-2002	Non-response rate	The proportion of eligible observational units for which no information could be obtained.
DQI-2003	Refusal rate	The proportion of eligible individuals who refuse to give the information sought.
DQI-2004	Drop-out rate	The proportion of all participants who only partially complete the study and prematurely abandon it.
DQI-2005	Missing due to specified reason	Information in a data collection that is missing due to a specified reason.
Consistency		
DQI-3001	Inadmissible numerical values	Observed numerical data values are not admissible according to the allowed ranges.
DQI-3002	Inadmissible time-date values	Observed time-date values are not admissible according to the allowed time and date ranges.
DQI-3003	Inadmissible categorical values	Observed categorical data values are not admissible according to the allowed categories.
DQI-3004	Inadmissible standardized vocabulary	Data values are not admissible according to the reference vocabulary.
DQI-3005	Inadmissible precision	The precision of observed numerical data values does not match the expected precision.
DQI-3006	Uncertain numerical values	Observed numerical values are uncertain or improbable because they are outside the expected ranges.
DQI-3007	Uncertain time-date values	Observed time-date values are uncertain or improbable because they are outside the expected ranges.
DQI-3008	Logical contradictions	Different data values appear in logically impossible combinations.
DQI-3009	Empirical contradictions	Different data values appear in combinations deemed impossible based on empirical reasoning.
Accuracy		
DQI-4001	Univariate outliers	Numerical data values deviate markedly from others in a univariate analysis.
DQI-4002	Multivariate outliers	Numerical data values deviate markedly from others in a multivariate analysis.
DQI-4003	Unexpected locations	Observed location parameters differ from expected location parameters.
DQI-4004	Unexpected shape	The observed shape of a distribution differs from the expected shape.
DQI-4005	Unexpected scale	Observed scale parameters differ from expected scale parameters.
DQI-4006	Unexpected proportions	Observed proportions differ from expected proportions.
DQI-4007	Unexpected association strength	The observed strength of an association deviates from the expected strength of the association.
DQI-4008	Unexpected association direction	The observed direction of an association (e.g. negative, positive) deviates from the expected direction.
DQI-4009	Unexpected association form	The observed form of an association (e.g. linear, quadratic, exponential...) deviates from the expected form.
DQI-4010	Inter-Class reliability	Differences between classes (e.g. examiners) when measuring the same or similar objects under specified conditions.
DQI-4011	Intra-Class reliability	Differences within classes (e.g. examiners) when measuring the same or similar objects under specified conditions.
DQI-4012	Disagreement with gold standard	Differences with a gold standard when measuring the same or similar objects under specified conditions.

The term “expected” refers to a test criterion as annotated in metadata fields

### Integrity

Integrity related analyses are guided by the question: Do all data comply with pre-specified structural and technical requirements? Addressing this as an independent step is necessary in any data quality assessment, because study data and metadata are often deficient. The three domains within this dimension address:

1. the structurally correct representation of data elements or data records within data sets (*structural data set error*), e.g. a mismatch of observed and expected number of data records;
2. the correspondence between multiple data sets (*relational data set error*), e.g. the appropriate integration of multiple study data sets; and
3. the correct representation of data values within data sets (*value format error*), e.g. a mismatch between the expected and observed data type.

Deficits at the *integrity* level may invalidate any findings at subsequent stages of data quality assessments and for any substantial scientific analyses*.* Assessments of metadata are confined to the integrity domain.

### Completeness

*Completeness* related assessments are guided by the question: Are the expected data values available? Results provide knowledge about the frequency and distribution of missing data. Two domains within *completeness* treat missing data differently. Within the “*crude missingness*” domain, any specific reasons that underlie missing data are ignored because missing data are often improperly coded and meaningful indicators must nevertheless be computable. A common example is the provision of system-indicated missing values only such as NA in R. This impedes inferences on why data values are not available without context information. In contrast, “*Qualified missingness*” makes use of coded reasons for missing data such as refusals, met exclusion criteria or any other reason. The use of such missing codes enables the valid computation of non-response or refusal rates [38].

Missing data occur at different stages of a data collection. Reasons for participants not entering a study (1: *unit missingness*) may be different from those prompting a participant to leave the study after initial participation (2: *longitudinal missingness*, e.g. drop-out). Further restraints may impede the conduct of a segment of the study, such as a specific examination (3: *segment missingness*, e.g. taking part in an ultrasound examination). Within segments, there may be a failure to fully collect information (4: *item missingness*, e.g. refusal to respond to a question). Different sets of actionable information may result at each of these stages, both at the level of data quality management and statistical analyses. Analysing missing data at the stages 1 to 3 should forego the assessment of *item missingness*.

### Correctness: consistency and accuracy

*Correctness* related analyses are guided by the question: Are data values free of errors? The first dimension, *consistency* comprises indicators that use Boolean type checks to identify inadmissible, impossible, or uncertain data values or combinations of data values. The domain *range and value violations* targets single data values that do not comply with allowed data values or value ranges [39]. The second domain, *contradictions* examines impossible or improbable combinations of multiple data values.

In contrast, indicators within the *accuracy* dimension use diverse statistical methods to identify unexpected data properties. Its first domain, *unexpected distributions* targets discrepancies between observed and expected distributional characteristics, e.g. the violation of an expected normal distribution. The second domain, *unexpected associations*, assesses discrepancies between observed and expected associations. The third domain, *disagreement of repeated measurements,* targets the correspondence between repeated measurements of the same outcome, for example related to the precision of measurements, or the correspondence with gold standard measurements.

### Implementations

Various methods exist to compute data quality indicators. For example, different approaches are available to calculate response rates [38] or to assess outliers [40, 41]. *Implementations* describe the actual computation of data quality indicators. They can be tailored to specific demands of data quality assessments and may summarize results from different indicators. *Implementations* may therefore be linked to any level of the data quality framework hierarchy, for example to provide overall estimates of data quality for some dimension. Changes of *implementations* do not constitute a modification of the data quality concept.

### Descriptors

Results of data quality assessments should be available in machine-readable format. This is a necessary precondition for automated processing and subsequent aggregation of results. Yet, not all data-quality-related information may be expressed in a machine-readable format. For example, histograms or smoothed curves [42] may provide important insights in addition to a statistical test of some assumption about a distribution or association. However, the detection of a data quality issue based on graphs relies on the implicit knowledge of a person inspecting the results. Such output without a machine-readable metric is named a *descriptor*. *A*ll descriptive statistics are *descriptors* as well. To consider a sample mean as being problematic without an explicit rule-based assessment relies on implicit knowledge. *A single descriptor* may provide information for different indicators, as there are various possible interpretations. For example, a scatterplot may serve to identify outliers but also to detect unexpected associations and distributional properties.

### Data quality and process variables

Data are collected over time, possibly at different sites, by different examiners using diverse methods. Ambient conditions may vary. Such sources of variability, coded as process variables [43], may affect measurements and result in data quality issues. Unexpected association of statistical parameters with process variables may constitute novel data quality problems and can be related to almost all data quality indicators. An example of high practical relevance are examiner effects (*indicator*: unexpected location, Table 2; implementation: examiner effects - margins, Table 3). Another example are time trends in the data. Such associations with process variables should routinely be targeted.

**Table 3:** Example R-Functions and their Links to The Data Quality Framework

R-function name	Implementations within the function	Linked with the following indicators
pro_applicability_matrix()	Checks the correspondence of study data with the metadata and accessibility to files. Each study data variable is examined regarding the data type and cross-checked with the specified data type in the metadata.	Unexpected data elements; data type mismatch
com_unit_missingness()	Evaluates on the level of entire observational units whether all measurements are missing.	Missing measurements (Unit level)
com_segment_missingness()	Evaluates whether all associated measurements at the level of study segments (e.g. single examinations or instruments) are missing for an observational unit. A pattern plot is provided as a *descriptor*.	Missing measurements (Segment level);
com_item_missingness()	Examines for each variable of the study data the amount and type of missing data according to specified missing/jump codes, including a count of data fields without any data entry like NA in R.	Missing measurements (Item level); specific missingness; uncertain missingness status
con_limit_deviations()	Assesses limit deviations, with regards to inadmissible and improbable values and counts deviations above/below the specified thresholds. Limits may comprise hard limits to identify inadmissible values, soft limits to identify improbable values, and detection limits which refer to a censoring based on the properties of the measurement devices used.	Inadmissible numerical values; inadmissible time-date values; uncertain numerical values; uncertain time-date values
con_inadmissible_categorical()	Compares the match of single data values with admissible categories, summarizes observed vs. expected data values and counts the violations.	Inadmissible categorical values
con_contradictions()	Compares two data values of the same observational unit by using one of 16 logical comparisons. Counts the number of contradictions.	Logical contradictions; empirical contradictions
acc_distributions()	Creates distributional plots (bar or histogram) for numerical measurements (float, integer). If a grouping variable is provided, stratified empirical cumulative distribution functions (ecdf) are plotted as well [20].	Indicators within the *unexpected distributions* domain
acc_univariate_outlier()	Computes distributional characteristics of numerical measurements (e.g. mean, standard deviation, skewness) and applies four different rules to identify univariate outliers, e.g. Tukey, Hubert, and six sigma [44–46]. Counts the number of outliers and indicates the direction (low/high).	Univariate outliers
acc_multivariate_outlier()	Computes the Mahalanobis distance of at least two variables and counts the number of extreme measurements. In a heuristic approach outlier identification is based on applying simple univariate rules [44–46] on the Mahalanobis distance to reduce computational costs.	Multivariate outliers
acc_shape_or_scale()	Tests the observed distribution of measurements against predefined distributional assumption (normal, gamma, uniform). Deviations from expected distributions are visualized using the idea of rootograms [44, 47].	Unexpected shape parameter; unexpected scale parameter
acc_end_digits()	Computes preferences of manually collected data, i.e. the preference of end digits. The functions assume a uniform distribution of end digits and applies a rootogram-like visualization [44, 47].	Unexpected shape
acc_margins()	Compares the marginal distribution of different classes (e.g. examiners, devices) using measurements adjusted for covariates (e.g. age, sex). Adjusted linear models, logistic regression or poisson-regression are used to model marginal means of continuous measurements, binary, and count data [48].	Unexpected location; unexpected proportion
acc_varcomp()	Computes the variance proportion explained by different classes (e.g. examiners, devices) in relation to the overall variance of the measurement. Depending on the data ANOVA or mixed effects models are applied [49, 50]	Unexpected location
acc_loess()	Computes and displays as a *descriptor* loess-smoothed trends of measurements across different classes over time. The raw measurements can be adjusted for covariates such as age or sex and the resulting residuals are smoothed over time using LOESS [42].	Indicators within the *unexpected distributions* domain, foremost unexpected location; unexpected proportion

### Using R and the data quality workflow

Data quality can be assessed using the R package dataquieR. Table 3 provides an overview of the applied computational and statistical methods. The use of dataquieR can be twofold: (1) all-at-once without an in-depth specification of parameters using the function dq_report() to create complete default reports or (2) step-by-step allowing for a detailed data quality assessment in a sequential approach. The first option checks the availability of metadata and applies all appropriate functions to the specified study data. A flexdashboard [51] is then generated which summarizes the results by data quality dimensions and variables.

**Fig. 3:**
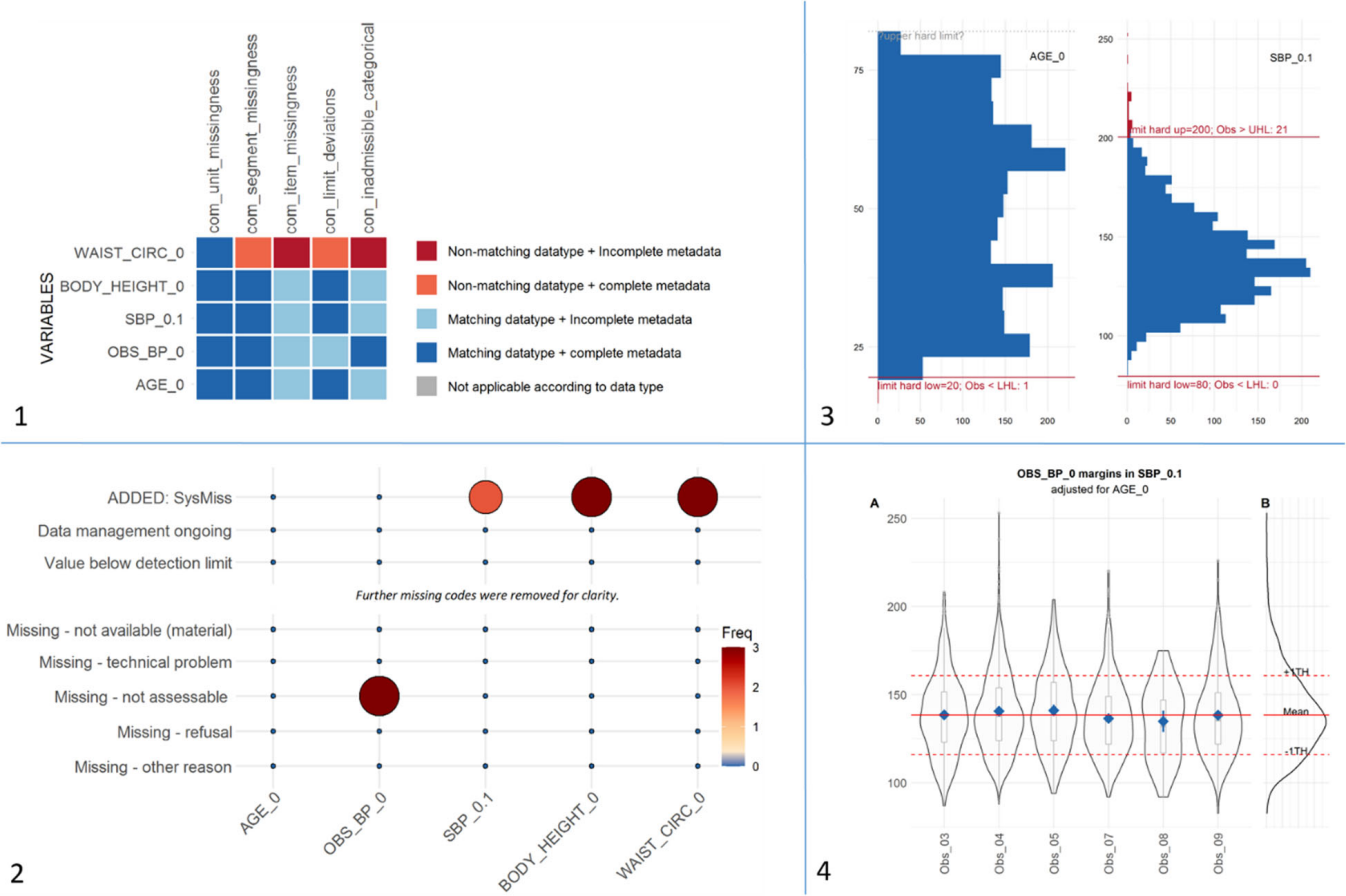
Example results using R dataquieR applied to SHIP data. 1: A heatmap-like plot to illustrate the applicability of data quality implementations based on an assessment of metadata and study data properties. 2: Histogram with illustrated range violations. 3: Illustration of missing values across different reasons for missing data. 4: Margins-plot to illustrate observer effects

In contrast, the sequential approach allows for specific parameter settings, changes to the output, corrections and modification of the data, and stratification according to additional variables. Examples of the step-by-step approach are shown in Fig. 3 using SHIP data. For the sake of clarity, only five variables (data elements) have been selected for display. First, the applicability of implementations to each data element was checked. Apparently, the data type of “waist circumference” did not comply with the data type specified in the metadata (Fig. 3, panel 1 top-left). After resolving this issue further data quality checks were conducted. Item missingness has been tabulated to provide insights about different reasons for missing data at this level (Fig. 3, panel 2 bottom-left). Afterwards the consistency of the data was examined with respect to limit deviations (Fig. 3, panel 3 top-right). Among the different applications addressing accuracy, the adjusted margins function compares mean values across observers to address examiner effects while adjusting for a for a vector of covariates (Fig. 3, panel 4 bottom-right). A commented example is available in the tutorial section of the webpage.

## Discussion

We provide a data quality framework for research data collections in observational health research, accompanied by software implementations in R. Data quality is addressed with regards to four core requirements: compliance with pre-specified structural and technical requirements (*integrity*), presence of data values (*completeness*), and absence of errors in the sense of, first, inadmissible data values, uncertain data values and contradictions (*consistency*) and second, unexpected distributions or associations (*accuracy*). To the best of our knowledge, this is the first data quality framework in the field that is accompanied by documented and freely available software code to compute indicators. A web page provides further guidance on all concepts and tools. The framework may promote harmonized data quality assessments and can be extended to accommodate other aspects of data quality and study types.

The framework was built from the perspective of “intrinsic data quality” [16] with requirements focussing on 1. processable data, 2. complete data, and 3. error-free data. The first dimension to target is *integrity,* as data quality assessments are a complex workflow where preconditions must be checked and reported first to safeguard the validity of subsequent results. *Integrity* in our framework resembles the *conformance* dimension in other approaches [8, 10], but focusses more narrowly structural requirements on data sets and data values. In practice, *integrity* checks often reveal recoverable issues. Additional data management processes may restore compliance with requirements, for example, by adding missing data structures.

In line with other approaches [6–8], *completeness* and *correctness* are the other main aspects of data quality. Both have been defined as core data quality constructs with regard to EHR data in the framework of Weiskopf et al. [9]. The stronger notion of *correctness* was preferred over p*lausibility* [8, 10] because the data generation in observational health research data collections is largely under the control of the researchers. This implies strong options to address errors during data collections and thereafter. We did not include the third core dimension by Weiskopf et al. [9], *currency,* which denotes whether “a value is representative of the clinically relevant time”. This aspect is considered to be of lesser importance in a research data collection from an intrinsic perspective.

Despite overlap with the TMF guideline [11, 14], Table 4, our data quality framework differs in several regards. The TMF-guideline focuses on registries while our framework focuses data collected for research purposes. Our framework is organized hierarchically, whereas there is no comparable structure in the TMF-guideline. TMF indicators correspond to different elements of our approach, ranging from data quality dimensions to implementations (Table 4). We cover all of the indicators classified as important [29] in the evaluation of the TMF-guideline with two exceptions: Compliance with operating procedures (TMF-1047) has not been included because information in standard operating procedures or study protocols is not available in an appropriate format for automated assessments. *Representativeness* (TMF-1048) can be formally targeted using indicators within the unexpected distributions domain to check observed sample properties against known population characteristics. It is however a matter of context-knowledge to interpret findings as a result of selection bias instead of measurement error. As such, representativeness is a contextual rather than an intrinsic aspect of data quality.

**Table 4:** Correspondence of TMF data quality indicators with the current data quality framework

TMFID	TMF name	Related in current framework to concept	Description of element type/ implementation in current framework
TMF-1001	Agreement with previous values	Disagreement of repeated measurements	Domain
TMF-1003	Consistency	Contradictions	Domain
TMF-1004	Certain contradiction/error	Certain contradictions	Indicator
TMF-1005	Possible contradiction/warning	Uncertain contradictions	Indicator
TMF-1006 TMF-1009 TMF-1010 TMF-1011 TMF-1052	Distribution of values Distribution of parameters recorded by the investigator Distribution of parameters recorded by the device Distribution of findings recorded by a medical reader Distribution of parameters between study sites	Unexpected location parameter Unexpected shape parameter Unexpected scale parameter Unexpected proportion	Indicator but TMF differentiates by the influencing factor while the current framework distinguishes by the statistical aspect.
TMF-1012	Missing modules	Unexpected data elements	An implementation that identifies missing modules within the indicator unexpected data elements
TMF-1013	Missing values in data elements	Missing values	Indicator
TMF-1014	Missing values in mandatory data elements	Missing values	An implementation that identifies mandatory data elements within the indicator missing values
TMF-1016	Data elements with value unknown etc.	Missing due to specified reason	Indicator (TMF targets a specific reason for missing value: unknown values)
TMF-1018	Outliers (continuous data elements)	Univariate outliers	Indicator
TMF-1019	Values that exceed the measurability limits	Inadmissible numerical values	Implementation within inadmissible numerical values
TMF-1021	Illegal values of qualitative data elements	Inadmissible categorical values	Indicator
TMF-1022	Illegal values of qualitative data elements used for the coding of missings	Inadmissible categorical values	An implementation that identifies inadmissible coding of missing modules within the indicator inadmissible categorical values
TMF-1023	Illegal values used for the coding of missing modules	Inadmissible categorical values	An implementation that identifies inadmissible coding of missing values within the indicator inadmissible categorical values
TMF-1024	Illegal values of qualitative data elements used for the coding of results exceeding measurability limits	Inadmissible categorical values	An implementation that identifies data elements with codes related to measurability limits within the indicator inadmissible categorical values
TMF-1029	Duplicates	Duplicates	Indicator
TMF-1030	Recruitment rate	Nonresponse rate	Indicator, the current framework uses the inverse. The link between both depends on the definition of recruitment and nonresponse rates
TMF-1031 TMF-1032	Refusal rate of investigations Refusal rate of modules	Refusal rate	Indicator with implementations at the level of examination modules or the entire study
TMF-1034	Drop-out-rate	Drop-out rate	Indicator
TMF-1042	Observational units with follow-up	Non-response rate (inverse at unit level, depending on implementation form)	Indicator
TMF-1043	Accuracy	Accuracy	Dimension
TMF-1046	Completeness	Completeness	Dimension

1) Included are TMF-indicators that have been classified as being at least important based on an empirical evaluation [29]. Two indicators with an important rating have not been included, “Compliance with procedural rule” (TMF-1047) and “Representativeness” (TMF-1048), as described in discussion

### Computation of data quality indicators

The necessity to develop software for data quality assessments has previously been acknowledged [8, 9]. Providing not only a theoretical framework but also the code to analyse data quality is important to facilitate homogeneous and transparent assessments across studies. This is also of relevance for the implementation of harmonized data quality assessments within complex research data infrastructures such as euCanSHare [52] or NFDI4Health, a federated research data infrastructure for personal health data [53]. Our implementations differ from most other available program codes [18–24] in that they are attached to a formal framework. To ensure the robustness of implementation, dozens of utility functions support their appropriate application in the background. Standards for the setup of metadata were defined to enable automated data quality checks [43] as well as for the programmed R routines to avoid heterogeneous programming code. This will facilitate extensions by other scientists. Further software implementations within the program Stata and a Java web-application [54] are currently being programmed.

### Data quality assessments in research

Data quality assessments must generate actionable information. While a study is carried out, the main aim is to detect and mitigate errors. After the end of a data collection, data quality assessments can be conceived as a specific aspect of initial data analysis [3], which aims “to provide reliable knowledge about the data to enable responsible statistical analyses and interpretation”. As such, the presented work also provides a framework for structuring initial data analysis.

Data quality assessments may be conducted locally at the sites of the respective data holders by using the software implementations above. Further transparency is possible if data quality related metadata is stored centrally in widely used metadata repositories. One example are the Opal and Mica [55] tools which are used, among others, in euCanSHare [52], Maelstrom [56], and NFDI4Health [53]. Another example is the Medical Data Models Portal, a meta-data registry for sharing and reusing medical forms [57]. Developments to host the necessary metadata in metadata repositories are currently ongoing.

Another aspect are intelligible metrics to communicate information about the achieved data quality, such as visual alerts. This has been implemented in the SHIP-project. Related standards could facilitate communication between scientists to leverage a common understanding of data quality. This goal is also pursued by the Data Nutrition Project [58]. Yet, the latter takes a different methodological approach and focusses primarily on the intended use of data, thus emphasizing contextual data quality [16], whereas we emphasize intrinsic data quality. Future extensions of our framework to cover contextual data quality may increase overlap. Vice versa, structural aspects of the framework and suggested workflow may be of relevance to guide other approaches.

Another goal is to improve the scientific reporting of studies and the further elaboration of guidance documents to cover aspects of data quality more extensively, such as for example by the EQUATOR (Enhancing the QUAlity and Transparency Of health Research) network [59] or the STRATOS (STRengthening Analytical Thinking for Observational Studies) initiative [60]. Furthermore, many funding bodies require data management plans but no system exists for the handling or reporting of data quality. Standardized data quality reports may accompany both, final reports and scientific papers to provide transparent insights into data properties and study success. As a necessary precondition for harmonized data quality assessments, the improved management of metadata would contribute to a better compliance with FAIR (**F**indable, **A**ccessible, **I**nteroperable and **R**eusable) data principles [61].

### Limitations and outlook

The presented data quality framework does not cover all aspects of “fitness for use” (ISO 8000) as contextual aspects have not been taken into account. For example, a single missing data value due to a technical error may trigger corrective actions during data collection but may not affect statistical analyses. Thresholds for critical amounts of missing data depend on the methods and aims of a statistical analysis plan [62]. Even without data quality issues at the intrinsic level some data set may prove unfit for the study of a research question because of issues such as an insufficient number of events if the main outcome is a time-to-event variable.

While the defined set of indicators suffices to address a wide range of data quality issues further expansions will be necessary. For example, speaking of non-response rate in studies without a clearly defined sampling frame may not be appropriate and additional indicators need to be added [38]. The framework currently also does not address specific demands arising from special data sources such as omics or medical imaging.

Indicators make no assumptions about the underlying reasons for data quality issues. It is up to the scientist or data manager to make causal decisions, for example on the presence of some type of bias [63]. This in turn relies on the study design being well-documented and the study being conducted accordingly [64, 65].

We defined indicators that are statistically computable in an automated workflow, using a set of study data and metadata. Therefore, we did not address approaches of source data verification. To avoid lengthy computational times, in some cases heuristic statistical methods have been favoured over ones that are more sophisticated.

The functionality of R code is supported by versatile and numerous utility function to mitigate user errors. Nonetheless, this code relies on the existence of sufficient metadata and metadata itself may constitute a gateway for data quality issues. Any user must comprehend the framework and the conventions underlying the definition of metadata. Because the handling of study data varies greatly across studies, interoperability issues may arise, and the provision of interfaces to facilitate data transfer will be an important future extension of our work. Therefore, an alignment of data quality related metadata with standards for information exchange such as HL7 FHIR [66] and common data models to enable data quality assessments without additional efforts in a harmonized fashion across data sets is a main objective [53, 67].

We have sketched application scenarios of data quality assessments during the research data life cycle, yet quantitative approaches to data quality are also of relevance in other areas of life. For example, data quality monitoring during study conduct shares structural similarities with quality improvement related activities in a hospital setting. Benchmarking is of relevance for production processes in industrial settings. Sustainable decision-making and innovation rests on the availability of data with adequate quality properties. Aspects of the outlined framework may be useful whenever data is collected for such purposes in a designed and controlled fashion. Yet, each application scenario has its specific requirements that likely require adaptions and extensions of this framework as well as the related software implementations.

## Conclusions

A data quality framework for research data collections in observational health research is provided with software implementations in the programming language R. The framework covers four core aspects of data quality: compliance with pre-specified formats and structures (*integrity*), the presence of data values (*completeness*), and errors in the data values in the sense of inadmissible or uncertain data values as well as contradictions (*consistency*) and unexpected distributions or associations (*accuracy*). R functions facilitate harmonized data quality assessments within and across studies in pursue of transparent and reproducible research. Applications of the framework and software implementations are not limited to research.

## Data Availability

The datasets generated and/or analyzed during the current study are available in the dataquieR repository on gitlab, https://gitlab.com/libreumg/dataquier/-/tree/master/inst/extdata

## Abbreviations

ANOVA: Analysis of variance
DQ: Data quality
DQI: Data quality indicator
ecdf: Empirical cumulative distribution functions
EHR: Electronic health records
EQUATOR: Enhancing the QUAlity and Transparency Of health Research
exdat: Examination date
FAIR: Findable, Accessible, Interoperable and Reusable
FHIR: Fast Healthcare Interoperability Resources
HL7: Health Level 7
IDA: Initial data analysis
ISO: International standards organization
LOESS: Locally estimated scatterplot smoothing
NA: Not applicable
sbp: Systolic blood pressure
SHIP: Study of Health in Pomerania
STRATOS: STRengthening Analytical Thinking for Observational Studies
TMF: Technology, Methods, and Infrastructure for Networked Medical Research
TMFID: TMF guideline identifier

## References

[1] Houston ML, Yu AP, Martin DA, Probst DY (2018). Defining and developing a generic framework for monitoring data quality in clinical research. AMIA Annu Symp Proc.

[2] Fortier I, Burton PR, Robson PJ, Ferretti V, Little J, L'Heureux F, Deschenes M, Knoppers BM, Doiron D, Keers JC, Linksted P, Harris JR, Lachance G, Boileau C, Pedersen NL, Hamilton CM, Hveem K, Borugian MJ, Gallagher RP, McLaughlin J, Parker L, Potter JD, Gallacher J, Kaaks R, Liu B, Sprosen T, Vilain A, Atkinson SA, Rengifo A, Morton R, Metspalu A, Wichmann HE, Tremblay M, Chisholm RL, Garcia-Montero A, Hillege H, Litton JE, Palmer LJ, Perola M, Wolffenbuttel BH, Peltonen L, Hudson TJ (2010). Quality, quantity and harmony: the DataSHaPER approach to integrating data across bioclinical studies. Int J Epidemiol.

[3] Huebner M, Le Cessie S, Schmidt CO, Vach W (2018). A contemporary conceptual framework for initial data analysis. Observ Stud.

[4] Maelstrom guidelines. https://www.maelstrom-research.org/page/maelstrom-guidelines. Accessed 25 Mar 2021.

[5] Arts DG, De Keizer NF, Scheffer GJ (2002). Defining and improving data quality in medical registries: a literature review, case study, and generic framework. J Am Med Inform Assoc.

[6] Stausberg J, Nasseh D, Nonnemacher M (2015). Measuring data quality: a review of the literature between 2005 and 2013. Stud Health Technol Inform.

[7] Weiskopf NG, Weng C (2013). Methods and dimensions of electronic health record data quality assessment: enabling reuse for clinical research. J Am Med Inform Assoc.

[8] Lee K, Weiskopf N, Pathak J (2017). A framework for data quality assessment in clinical research datasets. AMIA Annu Symp Proc.

[9] Weiskopf NG, Bakken S, Hripcsak G, Weng C (2017). A Data Quality Assessment Guideline for Electronic Health Record Data Reuse. EGEMS (Wash DC).

[10] Kahn MG, Callahan TJ, Barnard J, Bauck AE, Brown J, Davidson BN, Estiri H, Goerg C, Holve E, Johnson SG (2016). A Harmonized Data Quality Assessment Terminology and Framework for the Secondary Use of Electronic Health Record Data. EGEMS (Wash DC).

[11] Nonnemacher M, Nasseh D, Stausberg J (2014). Datenqualität in der medizinischen Forschung: Leitlinie zum Adaptiven Datenmanagement in Kohortenstudien und Registern.

[12] European Centre for Disease Prevention and Control (2014). Data quality monitoring and surveillance system evaluation – A handbook of methods and applications.

[13] Warwick W, Johnsona S, Bonda J, Fletchera G, Kanellakisa P (2015). A framework to assess healthcare data quality. Eur J Soc Behav Sci.

[14] Stausberg J, Bauer U, Nasseh D, Pritzkuleit R, Schmidt CO, Schrader T, Nonnemacher M (2019). Indicators of data quality: review and requirements from the perspective of networked medical research. MIBE.

[15] Nonnemacher M, Weiland D, Stausberg J (2007). Leitlinie zum adaptiven Management von Datenqualität in Kohortenstudien und Registern Berlin: Medizinisch Wissenschaftliche Verlagsgeselschaft.

[16] Wang RY, Strong DM (1996). Beyond accuracy: what data quality means to data consumers. J Manag Inf Syst.

[17] Keller S, Korkmaz G, Orr M, Schroeder A, Shipp S (2017). The evolution of data quality: understanding the Transdisciplinary origins of data quality concepts and approaches. Annual Review of Statistics and Its Application.

[18] Kandel S, Parikh R, Paepcke A, Hellerstein JM, Heer J (2012). Profiler: Integrated statistical analysis and visualization for data quality assessment. Proceedings of the International Working Conference on Advanced Visual Interfaces: 2012.

[19] Golling T, Hayward H, Onyisi P, Stelzer H, Waller P (2012). The ATLAS data quality defect database system. The European Physical Journal C.

[20] Dasu T, Johnson T (2003). Exploratory data mining and data cleaning.

[21] De Jonge E, Van Der Loo M (2013). An introduction to data cleaning with R: statistics Netherlands Heerlen.

[22] Templ M, Filzmoser P (2008). Visualization of missing values using the R-package VIM. Reserach report cs-2008-1, Department of Statistics and Probability Therory, Vienna University of Technology.

[23] Comtois D. R package ‘summarytools’; 2016. https://CRAN.R-project.org/package=summarytools.

[24] Waljee AK, Mukherjee A, Singal AG, Zhang Y, Warren J, Balis U, Marrero J, Zhu J, Higgins PD (2013). Comparison of imputation methods for missing laboratory data in medicine. BMJ Open.

[25] Observational Health Data Sciences and Informatics (OHDSI). Data quality dashboard. https://data.ohdsi.org/DataQualityDashboard/. Accessed 25 Mar 2021.

[26] Kapsner LA, Kampf MO, Seuchter SA, Kamdje-Wabo G, Gradinger T, Ganslandt T, Mate S, Gruendner J, Kraska D, Prokosch H-U (2019). Moving towards an EHR data quality framework: the MIRACUM approach. Stud Health Technol Inform.

[27] Stausberg J, Bauer U, Nasseh D, Pritzkuleit R, Schmidt CO, Schrader T. Nonnemacher M: Indicators of data quality: review and requirements from the perspective of networked medical research. MIBE. 2019;15(1). (ePub). 10.3205/mibe000199.

[28] Chen H, Hailey D, Wang N, Yu P (2014). A review of data quality assessment methods for public health information systems. Int J Environ Res Public Health.

[29] Schmidt C, Richter A, Enzenbach C, Pohlabeln H, Meisinger C, Wellmann J, et al. Assessment of a data quality guideline by representatives of German epidemiologic cohort studies. GMS Med Inform Biom Epidemiol. 2019;15(1). (ePub). 10.3205/mibe000203.

[30] Richter A, Schmidt CO, Struckmann S. dataquieR: Data Quality in Epidemiological Research; 2021. https://CRAN.R-project.org/package=dataquieR.

[31] Development R, Core team (2020). R: a language and environment for statistical computing.

[32] Wickham H (2014). Advanced r: chapman and hall/CRC.

[33] Volzke H, Alte D, Schmidt CO, Radke D, Lorbeer R, Friedrich N, Aumann N, Lau K, Piontek M, Born G, Havemann C, Ittermann T, Schipf S, Haring R, Baumeister SE, Wallaschofski H, Nauck M, Frick S, Arnold A, Junger M, Mayerle J, Kraft M, Lerch MM, Dorr M, Reffelmann T, Empen K, Felix SB, Obst A, Koch B, Glaser S, Ewert R, Fietze I, Penzel T, Doren M, Rathmann W, Haerting J, Hannemann M, Ropcke J, Schminke U, Jurgens C, Tost F, Rettig R, Kors JA, Ungerer S, Hegenscheid K, Kuhn JP, Kuhn J, Hosten N, Puls R, Henke J, Gloger O, Teumer A, Homuth G, Volker U, Schwahn C, Holtfreter B, Polzer I, Kohlmann T, Grabe HJ, Rosskopf D, Kroemer HK, Kocher T, Biffar R, John U, Hoffmann W (2011). Cohort profile: the study of health in Pomerania. Int J Epidemiol.

[34] Loeffler M, Engel C, Ahnert P, Alfermann D, Arelin K, Baber R, Beutner F, Binder H, Brähler E, Burkhardt R (2015). The LIFE-adult-study: objectives and design of a population-based cohort study with 10,000 deeply phenotyped adults in Germany. BMC Public Health.

[35] Ahrens W, Siani A, Adan R, De Henauw S, Eiben G, Gwozdz W, Hebestreit A, Hunsberger M, Kaprio J, Krogh V (2017). Cohort Profile: The transition from childhood to adolescence in European children–how I. Family extends the IDEFICS cohort. Int J Epidemiol.

[36] Standards and Tools for Data Quality Assessment in Epidemiological Studies. https://dfg-qa.ship-med.uni-greifswald.de/. Accessed 25 Mar 2021.

[37] Patrick RL (1980). Data quality indicators and their use in data base systems.

[38] The American Association for Public Opinion Research (2016). Standard Definitions: Final Dispositions of Case Codes and Outcome Rates for Surveys.

[39] Brown J, Kahn M, Toh S (2013). Data quality assessment for comparative effectiveness research in distributed data networks. Med Care.

[40] Aguinis H, Gottfredson RK, Joo H (2013). Best-practice recommendations for defining, identifying, and handling outliers. Organ Res Methods.

[41] Sunderland KM, Beaton D, Fraser J, Kwan D, McLaughlin PM, Montero-Odasso M, Peltsch AJ, Pieruccini-Faria F, Sahlas DJ, Swartz RH (2019). The utility of multivariate outlier detection techniques for data quality evaluation in large studies: an application within the ONDRI project. BMC Med Res Methodol.

[42] Cleveland WS, Devlin SJ (1988). Locally weighted regression: an approach to regression analysis by local fitting. J Am Stat Assoc.

[43] Richter A, Schössow J, Werner A, Schauer B, Radke D, Henke J, et al. Data quality monitoring in clinical and observational epidemiologic studies: the role of metadata and process information. MIBE. 2019;15(1). (ePub). 10.3205/mibe000202.

[44] Tukey JW. Exploratory data analysis. Reading, Mass: Addison-Wesley Pub. Co. 1977.

[45] Hubert M, Vandervieren E (2008). An adjusted boxplot for skewed distributions. Comput Stat Data Anal.

[46] Sedlack JD (2010). The utilization of six sigma and statistical process control techniques in surgical quality improvement. J Healthc Qual.

[47] Kleiber C, Zeileis A. Visualizing count data regressions using rootograms. Am Stat. 2016;70(3):296–303. 10.1080/00031305.2016.1173590.

[48] Lenth RV (2016). Least-squares means: the R package lsmeans. J Stat Softw.

[49] Verbeke G (1997). Linear mixed models for longitudinal data. Linear mixed models in practice.

[50] Fahrmeir L, Heumann C, Künstler R, Pigeot I, Tutz G (2016). Statistik: Der weg zur datenanalyse: Springer-Verlag.

[51] Iannone R, Allaire JJ, Borges B. flexdashboard: R Markdown Format for Flexible Dashboards. R package version 0.5.1.1. https://CRAN.R-project.org/package=flexdashboard.

[52] euCanSHare project. http://www.eucanshare.eu/. Accessed 25 Mar 2021.

[53] NFDI4Health. https://www.nfdi4health.de/. Accessed 25 Mar 2021.

[54] Schmidt CO, Krabbe C, Schössow J, Albers M, Radke D, Henke J (2017). Square^2^ - a web application for data monitoring in epidemiological and clinical studies. Stud Health Technol Inform.

[55] Doiron D, Marcon Y, Fortier I, Burton P, Ferretti V (2017). Software application profile: opal and Mica: open-source software solutions for epidemiological data management, harmonization and dissemination. Int J Epidemiol.

[56] Bergeron J, Doiron D, Marcon Y, Ferretti V, Fortier I (2018). Fostering population-based cohort data discovery: the maelstrom research cataloguing toolkit. PLoS One.

[57] Gessner S, Neuhaus P, Varghese J, Bruland P, Meidt A, Soto-Rey I, Storck M, Doods J, Dugas M (2017). The portal of medical data models: where have we been and where are we going?. Stud Health Technol Inform.

[58] The Data Nutrition Project. https://datanutrition.org/. Accessed 25 Mar 2021.

[59] Simera I, Moher D, Hirst A, Hoey J, Schulz KF, Altman DG (2010). Transparent and accurate reporting increases reliability, utility, and impact of your research: reporting guidelines and the EQUATOR network. BMC Med.

[60] Sauerbrei W, Abrahamowicz M, Altman DG, le Cessie S, Carpenter J, on behalf of the STRATOS initiative (2014). STRengthening analytical thinking for observational studies: the STRATOS initiative. Stat Med.

[61] Wilkinson MD, Dumontier M, Aalbersberg IJ, Appleton G, Axton M, Baak A, Blomberg N, Boiten JW, da Silva Santos LB, Bourne PE (2016). The FAIR guiding principles for scientific data management and stewardship. Sci Data.

[62] Rubin DB, Little AH (2020). Statistical analysis with missing data.

[63] Grimes DA, Schulz KF (2002). Bias and causal associations in observational research. Lancet.

[64] Schmidt CO, Krabbe CEM, Schossow J, Berger K, Enzenbach C, Kamtsiuris P, Schone G, Houben R, Meisinger C, Bamberg F (2018). Quality standards for epidemiologic cohort studies: an evaluated catalogue of requirements for the conduct and preparation of cohort studies. Bundesgesundheitsblatt, Gesundheitsforschung, Gesundheitsschutz.

[65] Hoffmann W, Latza U, Baumeister SE, Brunger M, Buttmann-Schweiger N, Hardt J, Hoffmann V, Karch A, Richter A, Schmidt CO (2019). Guidelines and recommendations for ensuring good epidemiological practice (GEP): a guideline developed by the German Society for Epidemiology. Eur J Epidemiol.

[66] HL7 FHIR. Documentation index. 2019. http://hl7.org/fhir/documentation.html. Accessed 25 Mar 2021.

[67] Huser V, Kahn MG, Brown JS, Gouripeddi R (2018). Methods for examining data quality in healthcare integrated data repositories. Pac Symp Biocomput.

